# The Passive Immunoprotective Activity Using Egg Yolk IgY Antibodies of Live or Inactivated *Aeromonas veronii* Against Major Pathogenic Bacteria (*A. veronii* and *A. hydrophila*) in Fish

**DOI:** 10.3390/vetsci12090831

**Published:** 2025-08-29

**Authors:** Jing Chen, Pan Cui, Huihui Xiao, Xiaoqing Wu, Juan Lu, Yong Liu, Xiang Liu

**Affiliations:** 1 Anhui Province Key Laboratory of Embryo Development and Reproductive Regulation, Fuyang Normal University, Fuyang 236041, China; 23211308@stu.fynu.edu.cn (J.C.); 23211320@stu.fynu.edu.cn (P.C.); 22211302@stu.fynu.edu.cn (H.X.); 201806024@fynu.edu.cn (X.W.); 2 Anhui Provincial Key Laboratory of Molecular Enzymology and Mechanism of Major Metabolic Diseases, Auhui Provincial Engineering Research Centre for Molecular Detection and Diagnostics, College of Life Sciences, Anhui Normal University, Wuhu 241000, China; 3 Rural Revitalization Collaborative Technology Service Center of Anhui Province, Fuyang Normal University, Fuyang 236041, China; 200107024@fynu.edu.cn

**Keywords:** IgY antibody, passive immunization, aquaculture, *Aeromonas veronii*, *Aeromonas hydrophila*

## Abstract

Egg yolk immunoglobulin Y (IgY) has significant application potential in aquaculture as passive immunotherapy against various bacterial infections owing to its capacity for large-scale and cost-effective production. In this study, IgY antibodies of live or inactivated *Aeromonas veronii* were generated by laying hens immunization. Subsequently, passive immune protection experiments of the two IgY antibodies were conducted on goldfish (*Carassius auratus*) infected with *A. veronii* and *Aeromonas hydrophila*. The results indicated that both live and inactivated bacteria IgY antibodies provided significant passive protection rates (*p* < 0.05). Furthermore, ELISA tests demonstrated that the two IgY antibodies, as well as the serum of *C. auratus*, interacted with *A. veronii* or *A. hydrophila* (*p* < 0.05) in vitro. The bacterial loads in the kidneys of *C. auratus* immunized with the two IgY antibodies were decreased (*p* < 0.05), and *C. auratus* phagocytes had enhanced phagocytic activity. The expression levels of antioxidant factors (SOD, CAT, GSH-Px) and inflammatory factors mRNA (TNF-α, IL-1β, IL-6, IL-8) were down-regulated (*p* < 0.05). Additionally, histopathological analysis indicated that the renal, splenic, and intestinal tissue structures remained intact, and the immunofluorescence confirmed that apoptosis and DNA damage factors of p53 and γH2A.X reduced (*p* < 0.05), respectively. Thus, the IgY antibodies of live and inactivated *A. veronii* exhibit passive immune-protective effects against different pathogenic bacteria in *C. auratus*. Further, inactivated *A. veronii* immunization causes less damage to laying hens than that of live bacteria, which aligns more closely with welfare standards for laying hens, and the IgY of inactivated *A*. *veronii* is anticipated as a cross-protection against *A. veronii* and *A. hydrophila* infections in aquaculture.

## Simple Summary

Egg yolk IgY antibody has application value in the development of aquaculture passive immunotherapy. In this research, the IgY antibodies against live or inactivated *Aeromonas veronii* were prepared. We found that the two IgY antibodies could activate non-specific immune activities in goldfish (*Carassius auratus*) and hold the passive and passive cross-protective abilities to *C*. *auratus* against major aquaculture pathogens. Further, inactivated *A. veronii* immunization causes less damage to laying hens than that of live bacteria, which aligns more closely with welfare standards for laying hens, and the IgY of inactivated *A*. *veronii* is anticipated as a vaccine candidate for bacterial infections in aquaculture.

## 1. Introduction

Aquaculture occupies a significant position in global agriculture and fisheries. It plays an irreplaceable role in providing animal protein, promoting economic growth, and ensuring food security [1]. With the continuous expansion of aquaculture, the threat from diseases caused by pathogenic bacteria is becoming increasingly severe. Common pathogenic bacteria in aquaculture include *Aeromonas veronii*, *A. hydrophila*, *Vibrio vulnificus*, *Pseudomonas fluorescens*, *V. parahaemolyticus*, *Edwardsiella tarda*, and *V. alginolyticus*. These pathogenic bacteria lead to large-scale mortality in aquatic animals, pollute aquaculture water bodies, and pose potential threats to human health, resulting in significant economic losses and resource wastage in the aquaculture industry [2,3]. *A. veronii*, a Gram-negative pathogenic bacterium commonly found in freshwater and seawater environments, can cause diseases such as septicemia, enteritis, and abscesses in fish and crustaceans. It can result in large-scale mortality and pose a significant threat to the aquaculture industry [4].

Currently, commonly employed methods for pathogen treatment in aquaculture encompass antibiotics, traditional Chinese medicine formulations, beneficial bacteria, and vaccines [5,6]. Antibiotics have been extensively utilized to treat bacterial diseases. However, prolonged use of these medications has resulted in significant issues, including drug resistance, drug residues, and environmental pollution [7,8]. Chinese medicine preparations, including the extract of garlic [9], scutellaria [10], honeysuckle [11], forsythia [12], astragalus [13], and *Houttuynia cordata* [14], have garnered attention due to their natural origins and low toxicity. However, the mechanisms of their action are complex, their effects are unstable, and they are challenging to apply in large-scale epidemics. Beneficial bacteria, such as lactic acid bacteria and *Bacillus subtilis*, inhibit the growth of pathogenic bacteria by regulating the micro-ecological environments of aquatic systems and animal intestines [13]. Nonetheless, their effectiveness is limited in sudden disease outbreaks.

Vaccines refer to biological products made from various pathogenic microorganisms for the prevention of pathogen infections [15], and can be implemented with various vaccine types, such as attenuated live, inactivated, and protein subunit vaccines. Currently used vaccines include the inactivated vaccine for *A. hydrophila* [16], the inactivated vaccine for *Vibrio alginolyticus*, and the outer membrane protein (OMP) vaccine [8], among others. However, most existing aquatic vaccines remain in the laboratory research phase, with few products available for practical application [5]. Therefore, it is essential to develop new, efficient, and environmentally friendly vaccines to combat aquaculture pathogens.

Passive immunotherapy approaches offer rapid immune protection by directly supplying exogenous antibodies to the host, such as immunoglobulin Y (IgY) from egg yolk, antiserum, and monoclonal antibodies [17]. This approach demonstrates significant advantages, particularly in addressing sudden or epidemic pathogen infections [18,19]. Mira et al. immunized hens with the APCH-p23 immunogen to prepare p23-IgY antibody, and found that it effectively reduced the duration of diarrhea caused by *Candida parapsilosis* in calves [20]. Liang et al. immunized hens with the nervous necrosis virus recombinant capsid protein and produced high-purity NNV-IgY antibody. Compared to a non-specific IgY, the NNV-IgY significantly reduced the appearance of vacuolar cytopathic effects in GS cells after incubation with the virus [21]. Additionally, IgY antibodies possess several advantageous characteristics, including their straightforward preparation, high yield, cost-effectiveness, and lack of residues. These qualities render them valuable for applications in sustainable animal breeding and the development of diagnostic kits [22,23,24]. Further, Liu et al. prepared the IgY antibodies against outer membrane proteins (PF1380 or ExbB) of *P. fluorescens* and found the two IgY hold immunoprotective activity to resist *P. fluorescens* and *A. hydrophila* in goldfish [19]. Xiao et al. found that the IgY of *Vibrio fluvialis* outer membrane protein VF08100 or VF14355 hold the immunoprotective abilities against *V. fluvialis* and *A. hydrophila* infections in fish [25,26]. However, there remains a notable deficiency in research related to their application in aquaculture. Especially, there is a lack of research on IgY that can resist major aquaculture pathogens (*A. veronii* and *A. hydrophila*).

**Figure 1:**
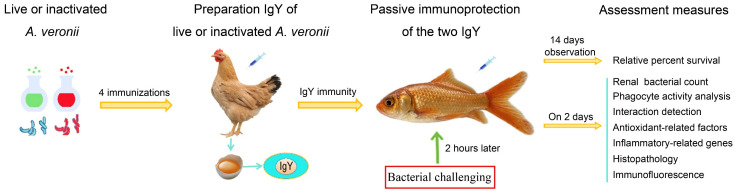
Experimental design.

In this study, we developed IgY antibodies against live or inactivated *A. veronii* by immunizing laying hens and used these IgY antibodies for the passive immunization in goldfish (*Carassius auratus*). Challenge experiments were conducted with *A. veronii* and *A. hydrophila*, and non-specific immune indices, protection rates, antioxidant and inflammatory responses, visceral tissue pathology, and immunofluorescence of apoptosis and DNA damage factors were assessed (Figure 1). The live or inactivated *A. veronii* IgY antibodies were evaluated for their ability to resist multiple bacteria, laying a theoretical foundation for the development of passive immunization vaccines in aquaculture.

## 2. Materials and Methods

### 2.1. Bacterial Strains

The microbiology laboratory of Fuyang Normal University in Anhui Province has preserved strains, including *A. veronii* ATCC35622, *A. hydrophila* ATCC7966, and *Staphylococcus aureus* ATCC6538.

### 2.2. Animals and Breeding

Twenty-week-old Laihong laying hens were purchased from Chongqing Tengxin Biological Technology Co., Ltd. (Chongqing, China), while *C. auratus* (20 ± 1.0 g) was obtained from the Fuyang Aquatic Animal Breeding Co., Ltd., Anhui Province (Fuyang, China). The breeding of Laihong laying hens and *C. auratus* were conducted as described by Xiao et al. [25].

### 2.3. Preparation of IgY Antibodies

IgY Antibodies of live and inactivated *A. veronii* were prepared as described by Liu et al. [19]. Briefly, *A. veronii* was cultured at 30 °C overnight, and was collected by centrifugation. A portion of the collected bacteria was inactivated by placing it in a 1% formaldehyde solution and 80 °C water for 90 min. The 400 μL of live or inactivated *A. veronii* (2 × 10^7^ CFU), and normal saline as blank IgY antibody group (nature control) were immunized to laying hens. Four immunization courses were conducted, with a 14-day interval between courses. After the fourth immunization, eggs were collected and stored at 4 °C refrigerator.

IgY antibody was purified from eggs with polyethylene glycol (PEG6000) as described by Ren et al. [27]. Briefly, after the egg yolk was separated with a yolk separator, the PEG6000 solution with a series of concentration (3.5, 8.5, and 12%) was added to the yolk. After being incubated at 25 °C for 30 min, the mixture was centrifuged at 12,000× *g* for 30 min. Then, the precipitate was dissolved in a phosphate-buffered saline (PBS) solution. Finally, the solution was dialyzed in a dialysis bag to obtain IgY antibody.

### 2.4. The Detection of In Vitro Interactions of IgY or C. auratus Serum with Pathogenic Bacteria

*A. veronii* or *A. hydrophila* were inoculated into LB medium at 30 °C. When the optical density at 600 nm (OD600) reached 1.0, the approximate cell concentration for *A. veronii* and *A. hydrophila* was 1 × 10^9^ and 9.9 × 10^8^ CFU/mL, respectively. The bacterial cells were harvested and transferred to the wells of an ELISA plate with 200 μL bacterial solution per well. The bacterial solution was incubated overnight at 4 °C for coating. Then, the plate wells were washed with PBS-Tween 20 (PBST) solution. Subsequently, a 5% skim milk solution was added to the wells for blocking, followed by incubation, typically at 37 °C for 1.5 h. After blocking, the wells were washed again. The serum from three *C. auratus* (immunized with IgY and challenged with bacteria) or the IgY antibodies were diluted in a series of gradients (dilution ratios of 1:200, 1:400, 1:800, 1:1600, 1:3200, and 1:6400) and added to each well for incubation at 37 °C for 2 h. After washing, a secondary antibody of Goat anti-chicken IgY (Sangon Biotech Co., Ltd., Shanghai, China) (dilution ratio of 1:1000) was added to the wells and incubated at 37 °C for 1.5 h. After the final incubation, the wells were washed again with PBST. Finally, a chromogenic solution was added and typically incubated at 37 °C for 10 min. A stop solution was then added to terminate the reaction, and the results were obtained using an enzyme-labeled instrument at a wavelength of 450 nm [28].

### 2.5. Median Lethal Dose (LD_50_) of A. veronii or A. hydrophila in C. auratus

The *C. auratus* were divided into two major groups for challenging with *A. veronii* or *A. hydrophila*, respectively. Each major group was further divided into four subgroups, with 10 fish in each subgroup. Each group of fish was intraperitoneally injected with a dose of 40 μL of blank IgY antibodies (40 μg). Two hours after the injection, the fish were intraperitoneally challenged with a dose of 40 μL of *A. veronii* (4 × 10^8^, 8 × 10^8^, 10 × 10^8^, 12 × 10^8^ CFU/fish) or *A. hydrophila* (1 × 10^9^, 2 × 10^9^, 4 × 10^9^, 8 × 10^9^ CFU/fish), respectively. Four days later, the LD_50_ of *A. veronii* or *A. hydrophila* was determined by calculating the bacterial dose at which half of the fish died. The results showed that the LD_50_ of *A. veronii* and *A. hydrophila* were 8 × 10^8^ CFU/fish and 4 × 10^9^ CFU/fish, respectively.

### 2.6. Passive Protection and Passive Cross-Protection Rate of IgY Antibodies

Passive immune protection rate was assessed as previously described [25]. Briefly, *C. auratus* was divided into 8 groups, with 15 fish in each group. The specific grouping is as follows: blank IgY group as nature control (NC), non-immunized IgY group (PBS solution) as negative control (NE-C), the live bacteria IgY antibody group, and the inactivated bacteria IgY antibody group were challenged with *A. veronii*, and four equivalent groups were challenged with *A. hydrophila*. Each group of fish was injected intraperitoneally with a dose of 40 μL of IgY antibodies (40 μg) or PBS solution. Two hours after the injection, the fish were intraperitoneally challenged with a dose of 40 μL of *A. veronii* (8 × 10^8^ CFU/fish) or *A. hydrophila* (4 × 10^9^ CFU/fish) according to preliminary lethal dose (LD_50_) determination experiment of fish bacterial challenge, respectively. The clinical symptoms were assessed by observation and recording as described previously [19,27,28]. Briefly, after immunizing to IgY and challenging with bacteria, the fish was observed and analyzed the clinical symptoms with continuous 14 days every 8 h. The clinical symptoms of fish include swimming vitality, epidermal bleeding, and abdominal swelling, as well as mortality. Relative percent survival (RPS) was calculated to evaluate the immune protective rate using the following formula: RPS (%) = (1 − [mortality rate of the experimental group%/mortality rate of the control group%]) × 100. The mortality rate of the experimental group was determined after its inoculation with live and inactivated bacteria IgY antibodies, while the mortality rate of the control group was based on the blank IgY group. All data were statistically analyzed using SPSS 19.0 software.

### 2.7. Renal Bacterial Count

*C. auratus* was immunized with live or inactivated *A*. *veronii* IgY antibodies and subsequently challenged with *A. veronii* and *A. hydrophila*. The control group received an injection of a blank IgY antibody prepared by immunization with normal saline for laying hens. To detect the possible presence of bacteria in fish kidney, the fish immunized with PBS solution without bacterial infection (*A. veronii* or *A. hydrophila*) was used as negative control. After 2 days, three kidney tissue samples were collected in each group under anesthesia. 50 mg of kidney per fish was weighed for subsequent testing. After thoroughly grinding, the kidney tissue was added 200 μL of physiological saline solution. Then, the kidney homogenates were implemented serial 10-fold dilutions with physiological saline solution, and 200 μL of diluent was spread onto selective culture media for cultivation overnight at 30 °C. Following incubation, the bacterial colonies on the medium were observed and counted. The inhibitory effect of IgY-antibody-mediated immunity on *A. veronii* or *A. hydrophila* infection was evaluated by calculating the total number of colonies [26]. All data were statistically analyzed using SPSS 19.0 software.

### 2.8. Analysis of the Phagocyte Phagocytic Activity

The phagocytic activity of phagocyte was assessed as previously described [29]. Briefly, *C. auratus* was immunized with live or inactivated *A*. *veronii* IgY antibodies, and the control group received a blank IgY antibody, and subsequently challenged with a dose of 40 μL of *A. veronii* (8 × 10^8^ CFU/fish) or *A. hydrophila* (4 × 10^9^ CFU/fish). After two days, three blood samples of each group were collected from the caudal vein under anesthesia using anticoagulant centrifuge tubes to obtain plasma. Meanwhile, *S. aureus* was cultured overnight at 30 °C. After collecting the bacteria by centrifugation, the *S. aureus* was fully suspended in a 1% formaldehyde solution and placed in an 80 °C water bath for 90 min to obtain inactivated *S. aureus*. Then, the concentration of *S. aureus* was adjusted to *OD*_600_ = 1.0 using a physiological saline solution. Subsequently, 0.2 mL of the blood was mixed thoroughly with 0.2 mL of inactivated *S. aureus* (2 × 10^7^ CFU/mL). The mixture was then incubated in a water bath at 25 °C for 60 min. Following incubation, the blood and inactivated bacterial mixture were placed onto a slide and evenly spread to create a blood smear. The blood smears were stained using a quick Giemsa staining kit (Sangon Biotech Co., Ltd., Shanghai, China) according to the kit’s operational instructions. The stained blood smears were observed and analyzed under a microscope (Leica, Wetzlar, Germany). Phagocytes were counted under the microscope, and the phagocytic percentage (*PP*%) was calculated using the following formula: *PP*% = (number of cells participating in phagocytosis among 100 phagocytes/100) × 100%. Additionally, the phagocytic index (*PI*%) was calculated as follows: *PI*% = (number of bacteria in phagocytes/number of cells participating in phagocytosis) × 100%. All data were statistically analyzed using SPSS 19.0 software.

### 2.9. Analysis of Antioxidant Factors

On day two after *C. auratus* was passively immunized to IgY and challenged with *A. veronii* or *A. hydrophila*, three sera of each group were collected from the caudal vein of the fish under anesthesia. The levels of antioxidant factors, including superoxide dismutase (SOD), catalase (CAT), and glutathione peroxidase (GSH-Px), were evaluated following the instructions provided by the detection kit (Sangon Biotechnology Co., Ltd., Shanghai, China).

### 2.10. mRNA Expression of Inflammatory Factors

The expression of inflammatory factor mRNA was assessed using real-time quantitative PCR. Briefly, head kidneys and spleens were collected under anesthesia on the second day following *A. veronii* or *A. hydrophila* challenge in *C. auratus*. The three tissues of each group were thoroughly homogenized in liquid nitrogen. RNA was extracted and converted into cDNA according to the manufacturer’s instructions (Takara, Beijing, China). qRT-PCR was conducted using the SYBR^®^ Green Premix kit (Takara, Beijing, China) and synthetic primers (Supplementary Table S1). Then, the ΔCt (cycle threshold change) was calculated by comparing the Ct value of the target gene with that of the internal reference gene (*gapdh*). Subsequently, the difference in ΔCt values between the experimental and control groups was used to derive the ΔΔCt. Finally, the 2^−(ΔΔCt)^ formula was applied to analyze mRNA expression [29]. All data were statistically analyzed using SPSS 19.0 software.

### 2.11. Tissue Pathological Analysis

On day two after three *C. auratus* of each group were immunized with IgY and infected with *A. veronii* or *A. hydrophila*, kidney, spleen, and intestinal tissues were collected under anesthesia and immersed in Davidson fixative and a 10% formaldehyde solution for 24 h for fixation. Following fixation, the tissues underwent a gradient ethanol dehydration process (70%, 80%, 90%, 95%, and 100% ethanol in sequence) and were treated with xylene for transparency twice, each time for 30 min. The transparent tissues were embedded in paraffin at 60 °C and then cut into thin slices with a thickness of 4 μm using a paraffin slicer. The tissue slices were placed onto slides and dried in a 60 °C oven for 3 h. Subsequently, the tissue slices were dewaxed using xylene, followed by dehydration with a gradient of ethanol, and then cleared again with xylene. Next, the slices were stained with hematoxylin and eosin (H&E). After staining, the slices were cleared once more with xylene. Finally, the slices were mounted with neutral resin and observed under an optical microscope (Leica, Wetzlar, Germany), capturing images as necessary [19].

Through histopathological observation, the damage quantitation score of kidneys and spleens were assessed as previously described in Rabb et al. [30]. Briefly, the high score indicates more severe necrosis of kidney and spleen tissues (up to 4 points). 0 points: normal kidney and spleen tissues; 1 point: less necrosis (<5% necrosis of kidney and spleen tissues); 2 points: mild necrosis (5–25% necrosis of kidney and spleen tissues); 3 points: moderate necrosis (25–75% necrosis of kidney and spleen tissues); 4 points: severe necrosis (>75% necrosis of kidney and spleen tissues). All data were statistically analyzed using SPSS 19.0 software.

Additionally, through histopathological observation, the damage quantitation score of intestinal tissues were assessed as previously described in Deng et al. [31]. Briefly, the high score indicates more severe necrosis of intestinal tissue tissues (up to 4 points). 0 points: the villous epithelium of intestinal tissue is intact; 1 point: mild villous edema, with epithelial collapse limited to the apical villi; 2 points: mild necrosis of the middle villi; 3 points: moderate necrosis of the middle villi with visible crypts; 4 points: severe necrosis of villi with disappearance of epithelial structure. All data were statistically analyzed using SPSS 19.0 software.

### 2.12. Renal Immunofluorescence Analysis

After *C. auratus* was passive immunization with live or inactivated *A*. *veronii* IgY antibodies, and blank IgY antibody (control), the fish were challenged with *A. veronii* or *A. hydrophila*, and kidney sections were prepared. The three renal tissue slides of each group were dewaxed in xylene and subsequently hydrated in ethanol using a decreasing concentration gradient. Following treatment with the antigen repair solution, an immunohistochemical pen was employed to delineate the tissue. A blocking solution of 50 μL (5%) bovine serum albumin (BSA) was added within the delineated area, and blocking was performed at room temperature for 1.5 h. After blocking, the slides were washed with PBST. Monoclonal antibodies against p53 or γH2A.X (dilution ratio 1:500) were then applied to the tissue and incubated overnight at 4 °C. Following a wash, a secondary antibody solution (dilution ratio 1:1000) was added and incubated at 37 °C for 1 h. After washing, the nuclei were stained with 4′,6-diamino-2-phenylindole (DAPI) at room temperature in the dark for 10 min, after which the slides were mounted, and images were captured using a fluorescence microscope (Leica, Wetzlar, Germany) [19]. All data were statistically analyzed using SPSS 19.0 software.

### 2.13. Statistical Analysis

All the experimental data were expressed as mean ± SD, and all experiments were repeated at least three times. The significant difference from the respective control in all experiments was assessed with one-way analysis of variance (ANOVA) followed by Tukey’s multiple comparison test using a software of Statistical Package for the Social Sciences 19.0 (SPSS19.0). Values of *p* < 0.05 were considered statistically significant [29].

## 3. Results

### 3.1. The Passive Protection and Passive Cross-Protection Rates of IgY Antibodies Against C. auratus

To determine the bacterial challenge dose, the LD_50_ of *A. veronii* and *A. hydrophila* was performed. The results showed that the LD_50_ of *A. veronii* and *A. hydrophila* were 8 × 10^8^ CFU and 4 × 10^9^ CFU (Supplementary Table S2), respectively. 2 mL of purified live or inactivated *A. veronii* IgY were obtained (Supplementary Figure S1) by the PEG6000 purification method, with a concentration of 1 μg/μL.

**Figure 2:**
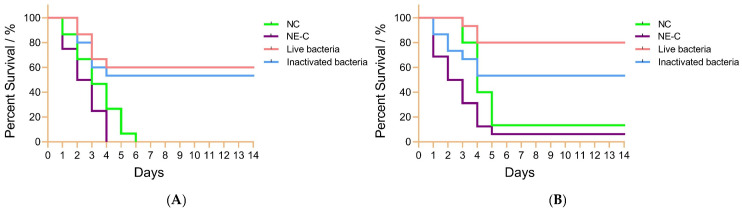
The survival rates of *C. auratus* infected with pathogenic bacteria. Panels (**A**,**B**) depict infections caused by *A. veronii* and *A. hydrophila*, respectively. Each group consists of 15 fish. NC represents blank IgY antibody as nature control. Live bacteria represent live *A. veronii* IgY. Inactivated bacteria represent inactivated *A. veronii* IgY. NE-C represents non-immunized IgY group (PBS solution) as negative control.

To investigate the differences in the protection rates of live and inactivated bacteria IgY antibodies against bacterial infections in fish, *C. auratus* was immunized with IgY antibodies and subsequently challenged with *A. veronii* and *A. hydrophila*, and the relative percent survival (RPS) was performed. Every 8 h. the mortality, physical characteristics, and activity of the fish were recorded and analyzed with a continuous recording for 14 days. The results indicated that *C. auratus* exhibited symptoms such as slow swimming, epidermal bleeding, and abdominal swelling, leading to a substantial number of fatalities. The mortality rate stabilized after six days (Figure 2). The RPS of live bacteria IgY antibody against *A. veronii* was 60% (*p* < 0.05), while that of inactivated bacteria IgY antibody was 53.33% (*p* < 0.05). Additionally, the RPS of live bacteria IgY antibody against *A. hydrophila* was 76.92% (*p* < 0.05), compared to 46.15% (*p* < 0.05) for inactivated bacteria IgY antibody (Table 1). There is no significant difference in the immune protection rate between these two IgY antibodies.

### 3.2. Determination of Bacterial Counts in the Kidney of C. auratus

**Figure 3:**
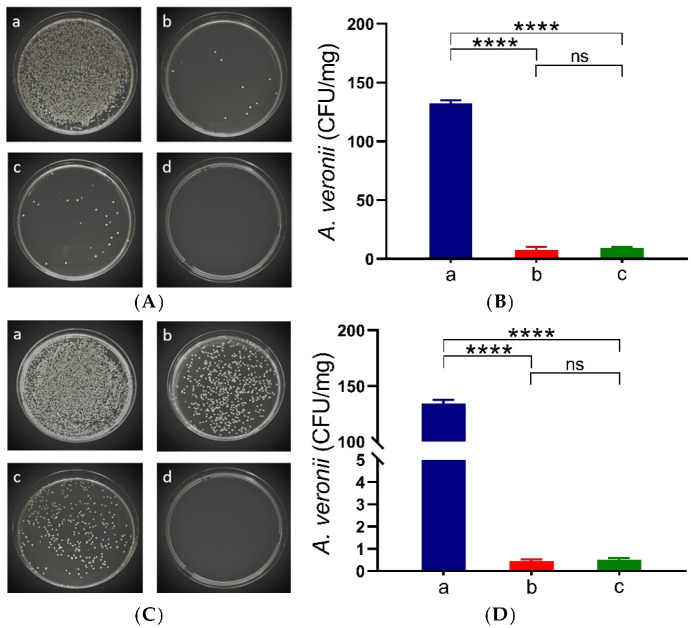
Kidney smears and bacterial counts in *C. auratus*. Panels (**A**,**C**) illustrate kidney bacterial colonies following infection with *A. veronii* and *A. hydrophila*, respectively. Panels (**B**,**D**) depict the colony counts for each infection. (**a**) Blank IgY antibody group (control). (**b**) Live bacteria IgY antibody group. (**c**) Inactivated bacteria IgY antibody group. (**d**) Fish immunized with PBS solution without bacterial infection (negative control). Data are presented as the mean ± SD (*n* = 3). Statistical significance compared to the blank IgY antibody group is denoted by **** *p* < 0.0001. ns indicates no significant difference, *p* > 0.05.

Kidney tissues were collected for culture plating two days after challenging the fish with *A. veronii* and *A. hydrophila* to evaluate the differences in bacterial counts in *C. auratus*. The results indicate that, compared to the control group, the bacterial counts in the kidneys of both the live and inactivated bacteria IgY antibody groups were significantly lower (*p* < 0.05) (Figure 3). There is no significant difference in the kidney bacterial count between these two IgY antibodies.

### 3.3. Detection of Phagocytic Activity of Phagocyte from C. auratus

To evaluate phagocyte phagocytosis in *C. auratus* immunized with IgY antibodies and subsequently challenged with pathogenic bacteria, we conducted cell phagocytosis experiments using the blood of *C. auratus*. The results indicate a significant increase in both the phagocytic index (*PI*) and the phagocytic percentage (*PP*) of phagocytes in the groups treated with live and inactivated bacteria IgY antibodies (*p* < 0.05) (Table 2). There is no significant difference between the two IgY.

### 3.4. Detection of Antioxidant-Related Factors (SOD, CAT, and GSH-Px) in the Serum of C. auratus

**Figure 4:**
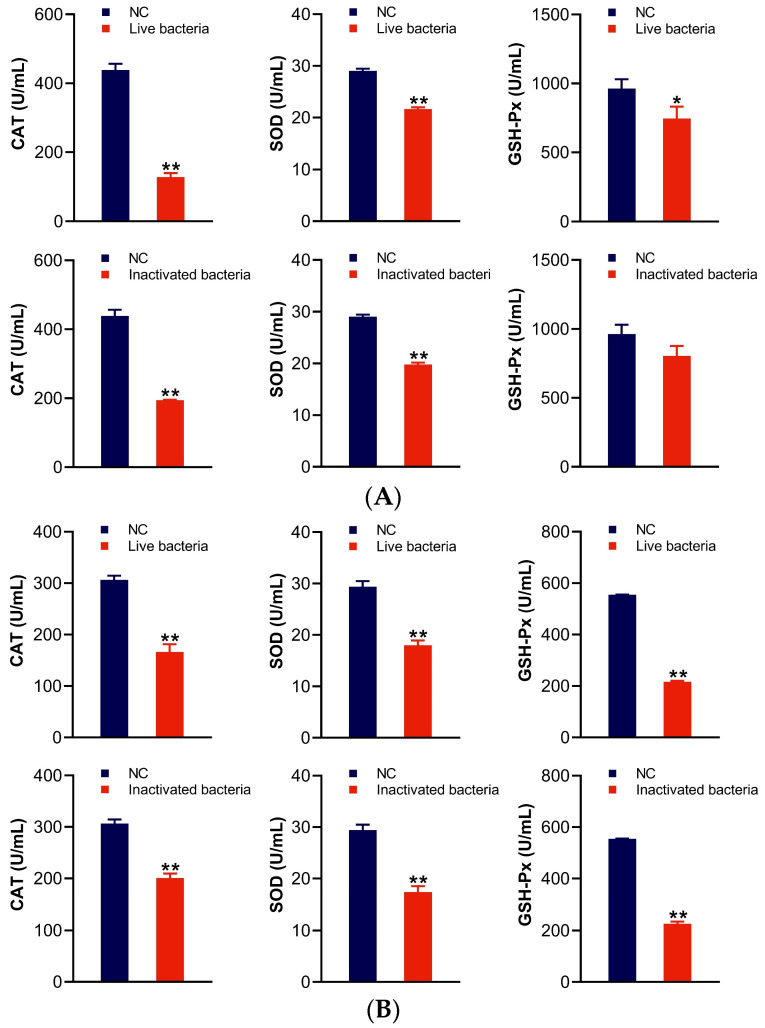
The activities of CAT, SOD, and GSH-Px in serum. Panels (**A**,**B**) correspond to *A. veronii* and *A. hydrophila*, respectively. Data are presented as the mean ± SD (*n* = 3). NC represents blank IgY group as nature control. Significant statistical comparisons with the control group are indicated by * *p* < 0.05 and ** *p* < 0.01.

The levels of antioxidant factors in the serum of *C. auratus* were evaluated on the second day following passive immunization with IgY antibodies and a subsequent bacterial challenge. The results demonstrate that, compared to the control group, the levels of most antioxidant-related factors (SOD, CAT, and GSH-Px) in the sera of the two IgY groups were significantly lower (*p* < 0.05) after the challenge with *A. veronii* and *A. hydrophila* (Figure 4). There is no significant differences between the two IgY.

### 3.5. Detection of the Expression of Inflammation-Related Genes in C. auratus

**Figure 5:**
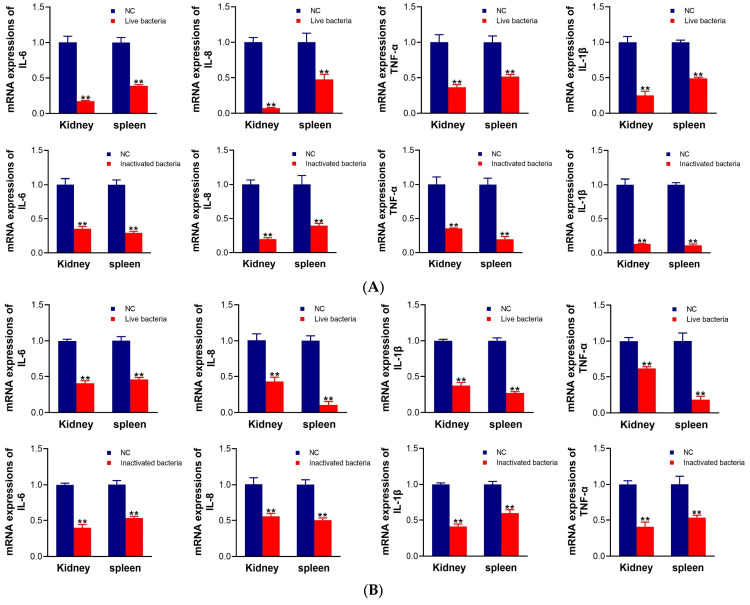
The mRNA expression levels of the inflammatory factors IL-6, IL-8, TNF-α, and IL-1β in tissues. (**A**,**B**) show the results of challenges with *A. veronii* and *A. hydrophila*, respectively. Data are presented as the mean ± SD (*n* = 3). The significant results compared with the control group: ** *p* < 0.01.

Following the passive immunization of *C. auratus* with IgY antibodies, the fish were challenged with *A. veronii* or *A. hydrophila*. The mRNA expression levels of inflammation-related genes (*il-6*, *il-8*, *tnf-α*, and *il-1β*) in the kidneys and spleens were subsequently evaluated. The results demonstrate that, compared to the control group, the mRNA expression levels of IL-6, IL-8, TNF-α, and IL-1β in the kidneys and spleens of the two IgY groups were significantly lower (*p* < 0.05) (Figure 5). There is no significant difference between the two IgY.

### 3.6. The Detection of In Vitro Interactions of IgY or C. auratus Serum with Pathogenic Bacteria

**Figure 6:**
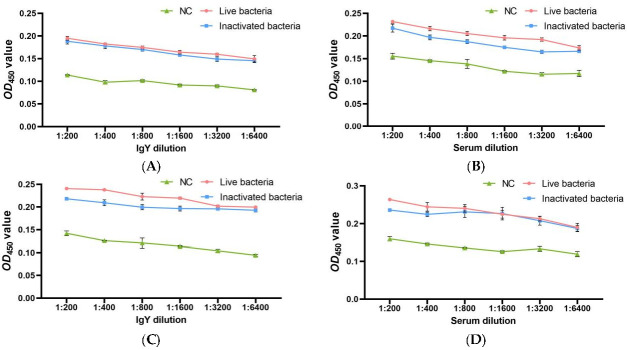
The recognition of IgY or *C. auratus* serum with pathogenic bacteria. Panel (**A**) depicts the recognition between IgY and *A. veronii*; (**B**) demonstrates the recognition between *C. auratus* serum (immunized with IgY and challenged with *A. veronii*) and *A. veronii*; (**C**) shows the recognition between IgY and *A. hydrophila*; and Panel (**D**) represents the recognition between *C. auratus* serum (immunized with IgY and challenged with *A. hydrophila*) and *A. hydrophila*. Data are presented as the mean ± SD (*n* = 3).

To investigate the in vitro interactions of live and inactivated bacteria IgY antibodies and bacteria, ELISA experiments were conducted. In the blank IgY group (NC), as the dilution of IgY increases, there is little change in *OD*_450_ values, and *OD*_450_ values are relatively small. This indicates that the blank IgY has no recognition effect on bacteria. Compared to the control group (NC), the two IgY antibodies could specifically bind to both *A. veronii* and *A. hydrophila*. Notably, the absorbance values gradually decreased with increasing antibody dilution; even at a dilution of 1: 6400, a significant interaction between IgY antibodies and pathogenic bacteria was still observed (*p* < 0.05) (Figure 6A,C). There is no significant difference between the two IgY.

Further, IgY antibodies were utilized to immunize *C. auratus*, and the fish were subsequently challenged with *A. veronii* and *A. hydrophila*. The interaction between the serum of *C. auratus* and the pathogenic bacteria was assessed. In the blank IgY group (NC), as the dilution of *C. auratus* serum increases, there is little change in *OD*_450_ values, and *OD*_450_ values are relatively small. This indicates that the serum of *C. auratus* immunized the blank IgY has no recognition effect on bacteria. In the two IgY groups, the absorbance decreased as the dilution of *C. auratus* serum increased. At a dilution of 1:6400, a significant interaction was observed between the serum of *C. auratus* passively immunized with the two IgY antibodies and *A. veronii* or *A. hydrophila* (*p* < 0.05), compared to the control group (*C. auratus* immunized with blank IgY) (Figure 6B,D). There is no significant difference between the two IgY.

### 3.7. Histopathological Observation of C. auratus Tissue Morphology

**Figure 7:**
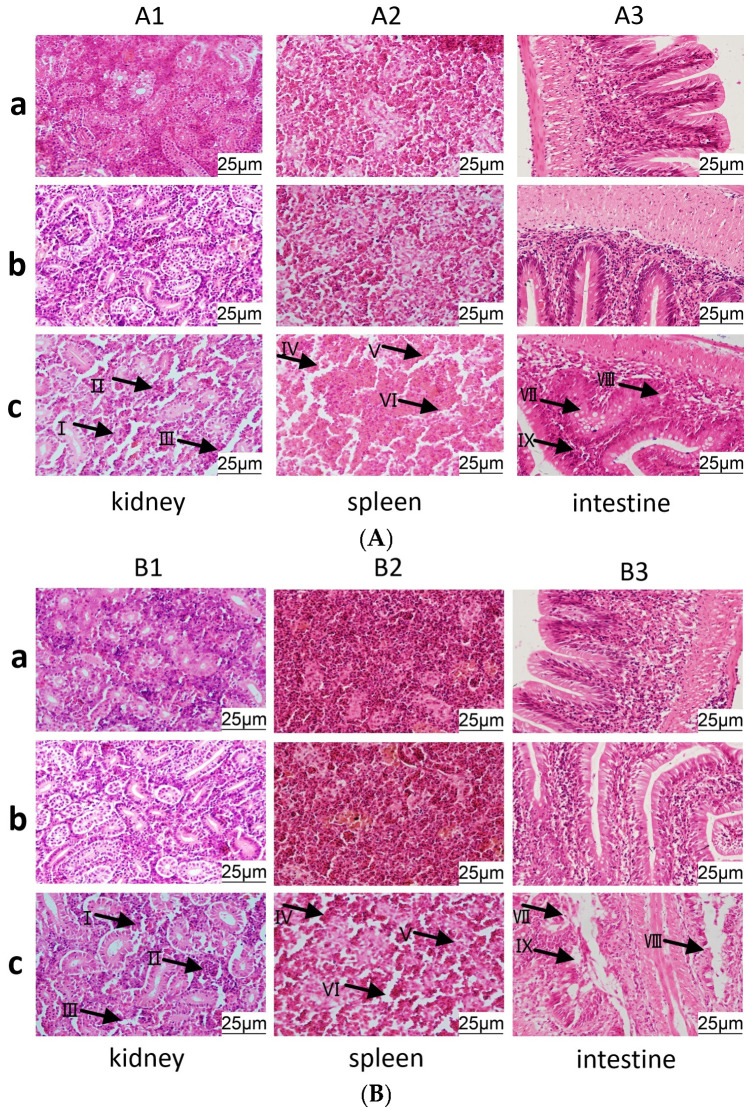
Pathological section maps of kidney, spleen, and intestinal tissues in *C. auratus*. (**A**,**B**) correspond to the challenge with *A. veronii* and *A. hydrophila*, respectively. (**a**) The group immunized with live bacteria IgY antibody. (**b**) The group immunized with inactivated bacteria IgY antibody. (**c**) The group immunized with blank IgY antibody. (**I**) Renal tubular apoptosis. (**II**) Glomerular degeneration. (**III**) Loose renal tissue structure. (**IV**) Decreased cell density in spleen. (**V**) Apoptosis of splenic cells. (**VI**) Incomplete spleen structure. (**VII**) Intestinal cell atrophy. (**VIII**) Incomplete intestinal structure. (**IX**) Atrophy of intestinal villi. Data are presented as the mean ± SD (*n* = 3).

To evaluate organ damage in *C. auratus* following infection with pathogenic bacteria, histopathological examinations were conducted on the kidneys, spleens, and intestines of fish immunized with live and inactivated bacteria IgY antibodies and subsequently infected with *A. veronii* and *A. hydrophila*. The results revealed that the renal tissue structure in the control group was loose and incomplete, with atrophy and degeneration observed in the glomeruli and renal tubules, accompanied by significant cell apoptosis (Figure 7(A1c,B1c)). Additionally, the splenic tissue structure was incomplete, with a marked reduction in spleen cell density and observable cell apoptosis (Figure 7(A2c,B2c)). Furthermore, inflammation has occurred in the intestines, and the lamina propria of intestinal villi showed atrophy and structural incompleteness and was also accompanied by intestinal cell apoptosis (Figure 7(A3c,B3c)). In contrast, the tissue structures of the kidneys, spleens, and intestines of both the live and inactivated bacteria IgY antibody groups remained intact and well defined, with no significant pathological damage observed (Figure 7). There is no significant difference between the two IgY.

Additionally, the results of damage quantitative scores showed that the scores of blank IgY group (NC) were higher than that of live or inactivated *A. veronii* IgY groups (*p* < 0.05) (Supplementary Figure S2). It indicates that the tissue damage of kidney, spleen and intestine in NC group is more severe than the two IgY groups. There is no significant difference between the two IgY.

### 3.8. Immunofluorescence Analysis on Kidney Tissues of C. auratus

**Figure 8:**
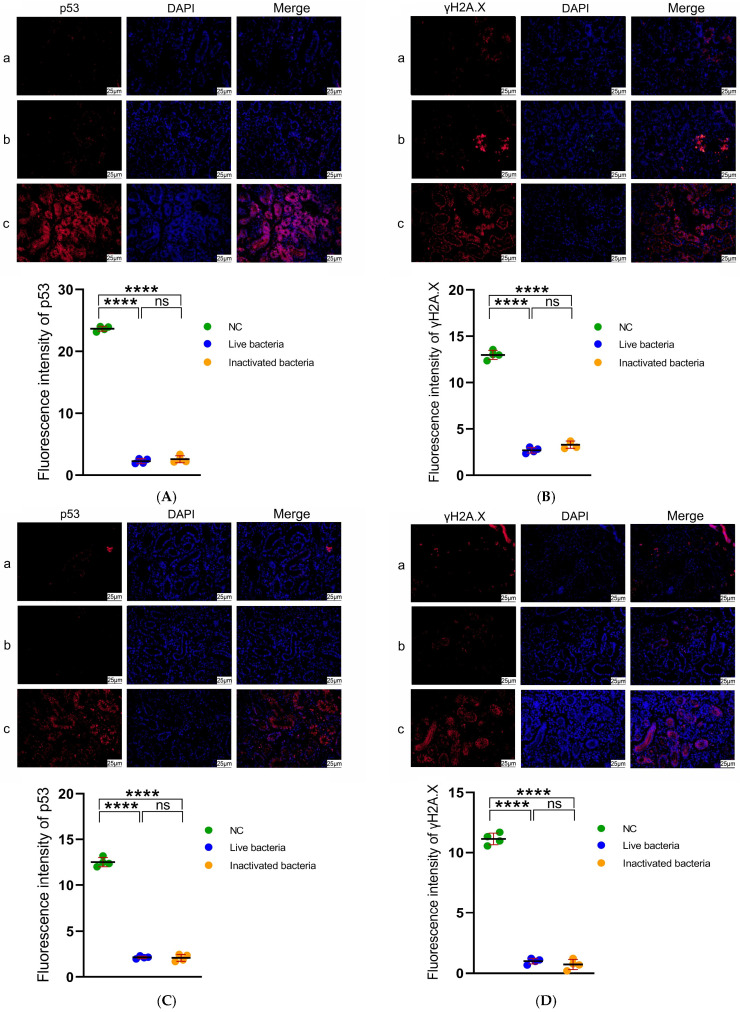
The immunofluorescence detection of p53 and γH2A.X proteins in the kidneys of *C. auratus*. Panels (**A**,**B**) depict the kidneys of *C. auratus* challenged with *A. veronii*, panels (**C**,**D**) present the kidneys of *C. auratus* challenged with *A. hydrophila*. Panels (**A**,**C**) show the expression levels of p53, panels (**B**,**D**) illustrate the expression levels of γH2A.X. (**a**) The group immunized with live bacteria IgY antibody. (**b**) The group immunized with inactivated bacteria IgY antibody. (**c**) The group immunized with blank IgY antibody. Data are presented as the mean ± SD (*n* = 3). Statistical comparisons with the control group reveal significant differences, indicated by **** *p* < 0.0001. ns indicates no significant difference, *p* > 0.05.

Immunofluorescence analysis was performed on the kidney tissues of *C. auratus* to evaluate apoptosis of kidney cells. Red fluorescence was employed to label the p53 and γH2AX proteins, while blue fluorescence was utilized for DAPI nuclear staining. The results indicate that, compared to the control group, the expression levels of p53 and γH2AX in both the live and inactivated bacteria IgY antibody groups were significantly lower (*p* < 0.05) (Figure 8). There is no significant difference between the two IgY.

## 4. Discussion

The multivalent IgY vaccine offers immune protection against multiple pathogenic bacteria. Its advantages include large-scale and cost-effective production, as well as cross-protection against various pathogenic bacteria [32,33]. The development of a multivalent IgY vaccine can enhance immune coverage, reduce the frequency of vaccine administration, and lower the associated costs. This makes it an ideal alternative to antibiotics in aquaculture [19,34]. Multiple studies have shown that polyvalent IgY vaccines have application potential in aquaculture [21,25,26,35]. This study utilized both live and inactivated strains of *A. veronii* to actively immunize laying hens, and eggs were collected to prepare a multivalent IgY vaccine for passive immunization. This vaccine was subsequently used to passively immunize *C. auratus*. The immune-protective effects of the vaccine against infections with various pathogens were assessed in *C. auratus*, thereby establishing a foundation for the development of aquatic vaccines.

The relative percent survival (RPS) serves as a critical indicator for assessing the efficacy of immune protection. By comparing the RPS of the immunized group and the control group, the immunoprotective effect of the drug can be determined [36,37,38]. The survival rate not only reflects the effectiveness of antibodies in controlling pathogen infections but also serves as an indirect measure of the host’s immune status and the extent of pathological damage. It is a crucial parameter for evaluating the efficacy of vaccines or antibodies [39]. Chen et al. (2025) prepared IgY antibody by immunizing hens with inactivated *V. vulnificus*. Following IgY treatment, zebrafish were challenged to *V. vulnificus*, and the results showed that the RPS in inactivated *V. vulnificus* IgY group was higher than that of control group [40]. Rizkiantino et al. (2023) immunized tilapia with the IgY of *Enterococcus faecalis*. Compared to the control group, the IgY group has a higher RPS to resist *Streptococcus* infection in fish [41]. In this study, we immunized *C. auratus* with both live and inactivated bacteria to evaluate the efficacy of IgY antibodies. The results indicate that the two IgY antibodies have passive and passive cross-protection abilities to resist the infection of *A. veronii* or *A. hydrophila* in *C. auratus*. Interestingly, the passive cross-protection rates of live bacteria IgY against *A. hydrophila* were 76.92%, which were higher than the direct protection against *A. veronii* (60%), although there was no significant difference between the two. *A. veronii* and *A. hydrophila* are both bacteria belonging to the genus *Aeromonas*, and the two bacteria may have homologous proteins or antigenic epitopes [27,28,29], which is consistent with this study’s results showing that live or inactivated *A. veronii* IgY provide passive immunoprotection to *A. veronii* or *A. hydrophila*. Additionally, this experiment was conducted according to the usual RPS test requirements with 15 fish per group [3,4,5] and lacked repeated experiments. Since only one tank per treatment was used, statistical analysis comparing RPS between treatments cannot be performed better. It is necessary to include replicate tanks in future experiments to allow valid statistical comparisons. These findings confirm that live and inactivated bacteria IgY antibodies provide passive immune protection against different bacterial strains, with no significant differences between the two types.

Non-specific immunity, also known as innate immunity, serves as the host’s primary line of defense against pathogen invasion. Commonly utilized evaluation indicators encompass cell phagocytosis, bacterial counts in visceral infections, in vitro detection of antigen–antibody interactions, and immune factor assessment [42]. Fajrinet al. (2023) aimed to explore the immunomodulatory potential of the ethanol extract (EE) and ethyl acetate fraction (EAF) derived from the rhizomes of *Curcuma heyneana* using mouse models. They found that, compared to the negative control, the white blood cell phagocytic index value was EE higher than EAF (*p* < 0.05) [43]. Bunnoy et al. (2023) developed a novel divalent mucosal adhesion nano-vaccine for the immersion vaccination of tilapia aimed at preventing francisellosis and columnaris disease. This vaccine significantly enhanced the phagocytic activity of tilapia cells [44]. Hayrapetyan et al. (2023) investigated the relationship between oral mucositis and oral bacterial counts in patients with head and neck cancer undergoing carbon ion radiotherapy [45]. In research on *Streptococcus equi*, Zhang et al. (2021) found that the srtA-5012 (R147G) mutant exhibited the lowest bacterial load in the lungs [37]. Bunnoy et al. (2023) indicate that adding antibodies to sample antigens and subsequently detecting the binding interaction rate between them can be utilized to identify the immune-protective effects of antibodies [44]. In this study, the phagocyte activity of *C. auratus* blood with live and inactivated *A. veronii* IgY was significantly increased. Concurrently, both live and inactivated bacteria IgY antibodies are effective in reducing bacterial loads in the kidneys, and the two IgY antibodies are specifically bound to the pathogen. Additionally, the serum of *C. auratus* passively immunized with the two IgY antibodies demonstrated specific binding to the pathogen. These findings suggest that both live and inactivated bacteria IgY antibodies, along with the serum from *C. auratus* immunized the two IgY, can effectively participate in antigen–antibody reactions. The antigen-IgY antibody complexes in vivo can enhance the recognition and phagocytosis of animal immune cells to eliminate the bacteria in the body [27,28]. These results indicate that the live and inactivated IgY antibodies activated the non-specific immunity of *C. auratus*.

The antioxidant factors in fish serum, including SOD, CAT, and GSH-Px, are crucial molecules for maintaining intracellular redox balance [46,47]. These factors effectively eliminate excessive reactive oxygen species, thereby safeguarding cells from oxidative damage, and the expression levels of antioxidant factors reflect the degree of oxidative stress [48,49]. Zhang et al. fed tilapia with the prepared specific IgY for 10 days, after which the fish were challenged with *Streptococcus agalactiae* [50]. The results indicated that the IgY antibody significantly reduced oxidative stress in tilapia. In this study, we observed a significant reduction in the levels of antioxidant factors (SOD, CAT, and GSH-Px) in the sera of *C. auratus* passively immunized with both live or inactivated bacteria IgY antibodies. This indicates that reduced SOD, CAT, and GSH-Px levels are more straightforwardly explained by decreased demand for these enzymes due to alleviated oxidative stress, maintaining stable oxidative reactions. Because this study found that after passive immunization with the two IgY and attacking bacteria, fish’s RPS increased, and renal bacterial count, inflammatory gene expression, and visceral tissue damage were all reduced. This finding suggests that the two IgY antibodies play a role in alleviating the oxidative stress response induced by pathogen infection.

The expression levels of inflammatory-related genes (*il-6*, *il-8*, *tnf-α*, and *il-1β*) in visceral tissue serve as crucial indicators for assessing the intensity of inflammatory reactions [51]. Inflammatory factors are signaling molecules released by the immune system in response to pathogen infections. Excessive expression of these factors can result in tissue damage and pathological changes. Zhu et al. found that resveratrol downregulated the levels of NF-κB, IL-1β, TNF-α, IL-8, GRP78, and CHOP in the ovaries of bighead carp at 6 and 9 months of age [52]. Shi et al. found that the carp that were not exposed to emamectin benzoate and microplastics, no inflammatory response was induced through the lysosome/ROS/ferroptosis pathway, and the expression of inflammation-related genes (*tnf-α*, *il-1β*) was significantly downregulated [53]. In this experiment, the expression levels of inflammation-related genes (*il-6*, *il-8*, *tnf-α*, and *il-1β*) in the kidneys and spleens of *C. auratus* from both the live and inactivated bacteria IgY antibody groups were significantly downregulated. This finding indicates that the two IgY antibodies can modulate the excessive inflammatory response triggered by pathogenic bacterial infections.

The relationship between antioxidant factors and the adaptive immune response is mainly reflected in their joint maintenance of the body’s defense ability [54]. Excessive reactive oxygen species (ROS) can attack the DNA and cell membrane of T/B cells, leading to decreased expression of MHC-II molecules and weakened antigen presentation activity, and decreased antigen recognition ability of T/B cells [55,56]. Antioxidant factors can eliminate ROS in the blood, inhibit lipid peroxidation, reduce oxidative stress damage to immune cells, and indirectly enhance the function of the immune system [57]. Additionally, inflammatory cytokines are messenger molecules in the immune system that help immune cells against infection and damage. When pathogens invade or tissues are damaged, immune cells (such as macrophages) secrete inflammatory cytokines, recruiting more immune cells (such as neutrophils and T cells) to the site of infection, and enhanced their ability to engulf and clear pathogens [58]. The immune system attacks pathogens through inflammatory cytokines, while in the later stages, anti-inflammatory cytokines suppress excessive reactions to prevent tissue damage caused by overexpression of inflammatory cytokines [59,60]. Therefore, the balanced expression of inflammatory cytokines is the core of maintaining the body’s immune ability. If the regulatory mechanism fails, inflammatory cytokines may trigger an “inflammatory storm”, causing chronic inflammation or immune diseases [61]. In this study, after passive immunization with live or inactivated A. veronii IgY and pathogenic bacteria challenge in fish, the expression of antioxidant and inflammatory factors remained low. It reflects that IgY can reduce the excessive adaptive immune response to maintain the balance of the immune response, reducing bacterial infection damage to fish.

Histopathology involves the examination of tissue structure and cellular morphology through microscopy, allowing for the assessment of the extent of damage inflicted by pathogen infections on host organs [62,63]. Tissue pathological sections of the kidneys, spleens, and intestines can directly reflect the pathological changes occurring in these organs during the infection process, including cell necrosis, inflammatory infiltration, and the destruction of tissue structure [64]. Liang et al. found that IgY antibody against capsid protein of nervous necrosis virus significantly inhibited the expression of the virus CP gene in the eyes and brains of fish, and effectively reduced tissue pathological damage by neutralizing the virus and alleviating the immune response [21]. Getnet et al. evaluated the water pollution situation by examining the pathological sections of the visceral tissues of Nile tilapia [65]. In this study, histopathological analysis revealed that the renal, splenic, and intestinal tissue structures of *C. auratus* passively immunized live or inactivated bacteria IgY remained intact, with no significant pathological damage observed after bacterial infection. In contrast, the control group exhibited severe damage, including glomerular and tubular atrophy, reduced spleen cell density, and atrophy of the intestinal villi. These findings indicate that IgY antibodies of live or inactivated bacteria can mitigate visceral tissue damage in *C. auratus*.

Immunofluorescence technology is capable of detecting the extent of apoptosis and DNA damage by labeling specific proteins, such as p53 and γH2A.X [66]. Ding et al. developed specific gene antibodies for zebrafish and employed these antibodies in slice immunofluorescence analysis to observe the expression changes in the corresponding genes [67]. Lv et al. also observed changes in the expression of p53 and γH2A.X using immunofluorescence techniques when evaluating the impact of nicotinamide phosphoribosyltransferase (NAMPT) inhibition on the radiosensitivity of malignant meningioma [68]. Kumar et al. found that, in hepatocyte-derived liver organoids (HepOrg) not exposed to aging inducers, the mRNA expression levels of γH2A.X, p53, and p21 decreased significantly [69]. In this research, the mRNA expression levels of γH2A.X and p53 in the kidney cells of *C. auratus* passively immunized live or inactivated IgY antibodies decreased, indicating a substantial reduction in DNA damage and cell apoptosis of kidneys. This finding validates the protective role of live or inactivated bacteria IgY antibodies in maintaining the integrity of host visceral tissues.

Altogether, this study found that live or inactivated *A. veronii* IgY can resist the infection of *A. veronii* or *A. hydrophila* in *C. auratus*. However, this research has limitations such as the limited pathogen scope and absence of long-term efficacy data, and it is necessary to conduct research on these issues in future work.

## 5. Conclusions

In this study, IgY antibodies against live or inactivated *A. veronii* were prepared, and a passive immunity model in *C. auratus* was utilized to evaluate the immune-protective effects against *A. veronii* and *A. hydrophila* infections. The experimental results indicate that live or inactivated bacteria IgY antibodies have passive cross-protection rates against bacterial infection. Furthermore, the two IgY antibodies can specifically bind to pathogenic bacteria in vitro, reduce the bacterial load in *C. auratus* kidneys, increase the phagocytic index of plasma, decreased the levels of serum antioxidant factors (SOD, CAT, and GSH-Px), and inhibited the mRNA expression of inflammatory-related genes (*il-6*, *il-8*, *tnf-α*, and *il-1β*) in kidney and spleen. Interestingly, the two IgY antibodies can maintain the integrity of renal, splenic, and intestinal tissue structures and reduce the expression of p53 and γH2A.X in kidney. Altogether, live and inactivated bacteria IgY antibodies can resist *A. veronii* and *A. hydrophila* infections in fish. Further, inactivated bacterial immunization aligns more closely with welfare standards for laying hens, and the IgY of inactivated *A*. *veronii* is promising potential as a cross-protective candidate against *A. veronii* and *A. hydrophila* infections in aquaculture.

## Figures and Tables

**Table 1:** The relative percent survival of IgY antibodies in *C. auratus*.

Bacteria	IgY Antibody	No.	Survival (no.)	Death (no.)	Cumulative Mortality Rate (%)	RPS (%)	RPS (*p*-Value)
*A. veronii*	NC	15	0	15	100	--	--
	NE-C	15	0	15	100	0	--
	Live bacteria	15	9	6	40.00	60.00 **	0.0007
	Inactivated bacteria	15	8	7	46.67	53.33 **	0.0003
*A. hydrophila*	NC	15	2	13	86.67	--	--
	NE-C	15	1	14	93.33	−7.68	0.5430
	Live bacteria	15	12	3	20.00	76.92 **	0.0002
	Inactivated bacteria	15	8	7	46.47	46.15 *	0.018

Note: RPS, relative percent survival. RPS (%) =1 − (vaccinated mortality%/non-vaccinated mortality%) × 100. Each group consists of 15 fish. NC represents blank IgY antibody as nature control. NE-C represents non-immunized IgY group (PBS solution) as negative control. * *p* < 0.05, ** *p* < 0.01 (compared with NC group).

**Table 2:** Results of the phagocytosis experiment on pathogenic bacteria in *C. auratus*.

Bacteria	Group (IgY)	*PP*%	*PI*%	*PP*% (*p*-Value)	*PI*% (*p*-Value)
Challenged with *A. veronii*	Control	31.53 ± 3.01 ^a^	70.27 ± 14.10 ^a^	--	--
	Live bacteria	60.80 ± 2.03 ^c^	130.40 ± 13.21 ^b^	0.0005	0.0004
	Inactivated bacteria	50.70 ± 1.27 ^b^	142.65 ± 8.34 ^b^	0.0004	0.0003
Challenged with *A. hydrophila*	Control	32.35 ± 1.36 ^a^	71.32 ± 7.36 ^a^	--	--
	Live bacteria	58.55 ± 0.89 ^c^	130.30 ± 4.70 ^b^	0.0006	0.0005
	Inactivated bacteria	52.38 ± 3.00 ^b^	139.50 ± 20.40 ^b^	0.0003	0.0004

Note: Data are presented as the mean ± SD (*n* = 3). The calculation methods are as follows: phagocytic percentage (*PP*%) = number of cells involved in phagocytosis among 100 phagocytic cells/100 × 100%. Phagocytic index (*PI*%) = number of bacteria in phagocytic cells/number of cells involved in phagocytosis × 100%. Labels a–c indicates statistically different groups (*p* < 0.05).

## Data Availability

All the data presented in the study are included in the article; further inquiries can be directed to the corresponding authors.

## Supplementary Materials

The following supporting information can be downloaded at https://www.mdpi.com/article/10.3390/vetsci12090831/s1, Figure S1: The purity of IgY antibodies were assessed by SDS-PAGE gel electrophoresis; Figure S2: The damage quantitative score of kidney, spleen and intestine; Table S1: Information on primers used for qRT-PCR; Table S2: The LD50 determination of *A. veronii* or *A. hydrophila* in *C. auratus*.
