# Supplementary material for: The Passive Immunoprotective Activity Using Egg Yolk IgY Antibodies of Live or Inactivated Aeromonas veronii Against Major Pathogenic Bacteria (A. veronii and A. hydrophila) in Fish
Source: Vet Sci. 2025 Aug 29;12(9):831. doi: 10.3390/vetsci12090831 (PMC12474119; doi:10.3390/vetsci12090831)
Supplement: Supplementary file 1 [file vetsci-12-00831-s001.zip › Supplementary Figure S1.pdf]

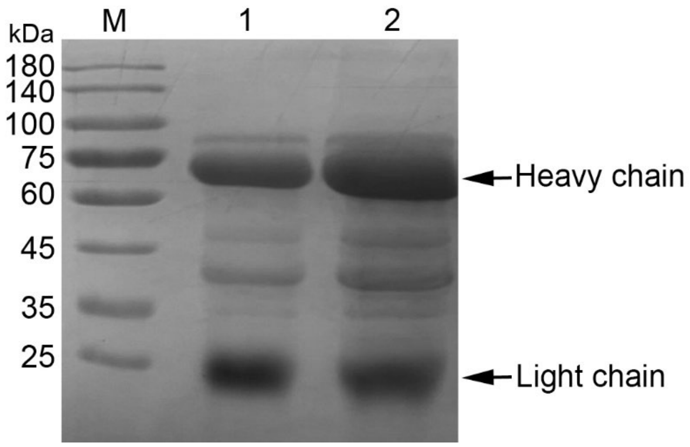

Supplementary Figure S1. The purity of IgY antibodies were assessed by SDS-PAGE gel electrophoresis. (M) protein marker. 1 and 2 represent live or inactivated *A. veronii* IgY.
