# Supplementary material for: The Passive Immunoprotective Activity Using Egg Yolk IgY Antibodies of Live or Inactivated Aeromonas veronii Against Major Pathogenic Bacteria (A. veronii and A. hydrophila) in Fish
Source: Vet Sci. 2025 Aug 29;12(9):831. doi: 10.3390/vetsci12090831 (PMC12474119; doi:10.3390/vetsci12090831)
Supplement: Supplementary file 1 [file vetsci-12-00831-s001.zip › Supplementary Figure S2.pdf]

A

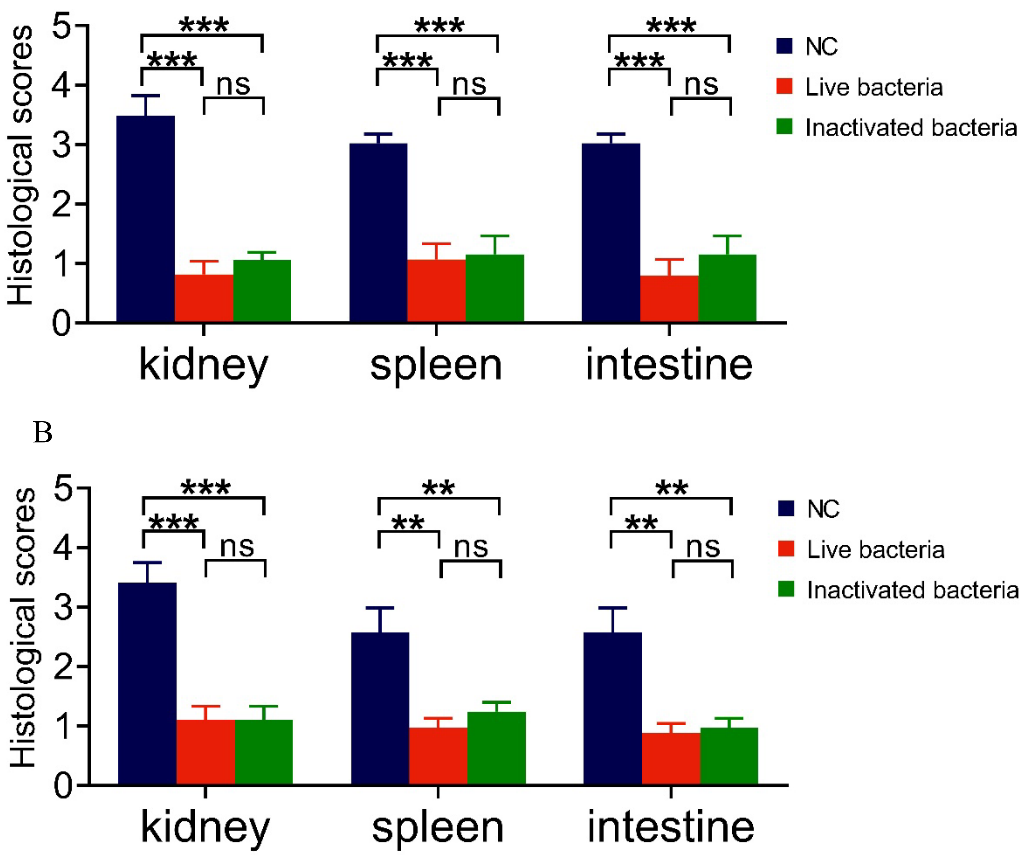

Supplementary Figure S2. The damage quantitative score of kidney, spleen and intestine. A and B correspond to the challenge with *A. veronii* and *A. hydrophila*, respectively. Data are presented as the mean ± SD (n = 3). Statistical comparisons with the control group (NC) reveal significant differences, indicated by **$p < 0.01$ and ***$p < 0.001$. ns indicates no significant difference, $p > 0.05$.
