# Supplementary material for: The Passive Immunoprotective Activity Using Egg Yolk IgY Antibodies of Live or Inactivated Aeromonas veronii Against Major Pathogenic Bacteria (A. veronii and A. hydrophila) in Fish
Source: Vet Sci. 2025 Aug 29;12(9):831. doi: 10.3390/vetsci12090831 (PMC12474119; doi:10.3390/vetsci12090831)
Supplement: Supplementary file 1 [file vetsci-12-00831-s001.zip › Supplementary Table S1.pdf]

Supplementary Table S1. Information on primers used for qRT-PCR.

Gene	Accession number	Forward primer (5′-3′)	Reverse primer (5′-3′)
il-6	XM_026289280.1	TCTCCTCAGACCCTCAGACG	CGTTTGGTCCCGTGTTTGAC
il-8	XM_026267284.1	GGAGTGCAGGCCACTGTTAG	ATCAGAAGCATGAAGGCGGA
tnf-α	EU069817.1	GGGCCACATCGTGATTCGTA	GCCTCCAGTGTAGCATGTGT
il-1β	AJ249136.1	TTCAGGAAAGAGACGGGCAC	GTCAGTTGGCACCTGGATCA
gapdh	XM_026284269.1	GATTTCAACGGGGATGTGCG	TCACACACACGGTTGCTGTA
