# Supplementary material for: The Passive Immunoprotective Activity Using Egg Yolk IgY Antibodies of Live or Inactivated Aeromonas veronii Against Major Pathogenic Bacteria (A. veronii and A. hydrophila) in Fish
Source: Vet Sci. 2025 Aug 29;12(9):831. doi: 10.3390/vetsci12090831 (PMC12474119; doi:10.3390/vetsci12090831)
Supplement: Supplementary file 1 [file vetsci-12-00831-s001.zip › Supplementary Table S2.pdf]

Supplementary Table S2. The LD_{50} determination of *A. veronii* or *A. hydrophila* in *C. auratus*.

**Bacteria**	**Bacterial dose (CFU)**	**No.**	**Death, no.**	**Survival, no.**	**ADR, %**
A. veronii	4×10^8	10	2	8	20
	8×10^8	10	5	5	50
	10×10^8	10	7	3	70
	12×10^8	10	10	0	100
A. hydrophila	1×10^9	10	1	9	10
	2×10^9	10	2	8	20
	4×10^9	10	5	5	50
	8×10^9	10	8	2	80

Note: ADR, accumulating death rate.
